# STROBE-causal machine learning for the human microbiome: systematic review on methodological innovations and validation frameworks

**DOI:** 10.3389/fmicb.2026.1705116

**Published:** 2026-03-25

**Authors:** Issam Khelfaoui, Wenxin Wang, Akram Ismael Shehata, Hicham Meskher, Mohammed F. El Basuini, Abdalla M. A. Mohamed, Mohamed F. Abouelenein, Houssem Eddine Degha, Mayada Alhoshy, Islam I. Teiba, Omnia Mahmoud, Seedahmed S. Mahmoud

**Affiliations:** 1 School of Public Health, Shantou University/Institute of Local Government Development, Shantou University, Shantou, China; 2 Guangdong Provincial Key Laboratory of Marine Biology, Shantou University, Shantou, China; 3 Institute of Marine Sciences, Shantou University, Shantou, China; 4 Department of Animal and Fish Production, Faculty of Agriculture (Saba Basha), Alexandria University, Alexandria, Egypt; 5 Department of Chemistry and Key Laboratory for Preparation and Application of Ordered Structural Materials of Guangdong Province, Shantou University, Shantou, China; 6 King Salman International University, South Sinai, Egypt; 7 Medical College, Shantou University, Shantou, China; 8 Department of Insurance and Risk Management, College of Business, Imam Mohammad Ibn Saud Islamic University (IMSIU), Riyadh, Saudi Arabia; 9 Department of Computer Science and Information Technologies, Faculty of New Technologies of Information and Communication, Kasdi Merbah Ouargla University, Ouargla, Algeria; 10 Independent Researcher, Alexandria, Egypt; 11 Botany Department, Faculty of Agriculture, Tanta University, Tanta, Egypt; 12 Department of Pharmacology, Shantou University Medical College, Shantou, China; 13 Department of Biomedical Engineering, College of Engineering, Shantou University, Shantou, China

**Keywords:** benchmarking, causal machine learning, human microbiome, microbiome-host interactions, reporting guidelines, reproducibility

## Abstract

The reproducibility crisis in causal microbiome research necessitates robust validation frameworks. Current studies often face inconsistent validation methods, limited interpretability, and a lack of standardized reporting, creating a gap in reliable causal inference. This systematic review evaluates over 60 peer-reviewed studies published between 2015 and 2024 to: (1) establish benchmarking standards leveraging synthetic data and biological plausibility assessments; (2) compare advanced causal machine learning (ML) methodologies, including Double/Debiased ML, Deep Instrumental Variables (Deep IV), and Directed Acyclic Graphs (DAGs), in their application to microbiome-host systems; and (3) propose the STROBE-CML (Strengthening the Reporting of Observational Studies in Epidemiology–Causal Machine Learning) guidelines to standardize reporting practices. We emphasize critical innovations such as federated validation pipelines and time-series causal discovery frameworks that address these gaps by facilitating scalable, privacy-preserving, and reproducible inference across heterogeneous cohorts. A decision support tool is introduced to guide researchers in selecting appropriate causal ML approaches based on data structure, research question, and computational constraints. By synthesizing methodological advances with rigorous validation paradigms, this review provides a roadmap for generating reliable, biologically interpretable, and clinically translatable causal claims in microbiome science.

## 1 Introduction

The human microbiome, once considered a passive commensal ecosystem, is now recognized as a central regulator of host physiology, immune function, and disease processes. Advances in high-throughput sequencing technologies have expanded our knowledge of microbial diversity and its associations with a wide range of health outcomes, including inflammatory bowel disease, cancer, obesity, and neurodegenerative disorders. Despite these advances, the overwhelming majority of reported microbiome–disease links remain correlational, offering limited insight into whether observed microbial shifts are causal drivers of disease or simply consequences of underlying host phenotypes. This limitation poses significant challenges for translating microbiome science into clinical and therapeutic applications. As the field matures, there is an urgent imperative to move beyond correlation and toward robust causal inference frameworks (Bogart et al., 2019; Khelfaoui et al., 2025).

The difficulty of establishing causality in microbiome research is compounded by the complex nature of microbiome data and fundamental epidemiological challenges. These data are inherently high-dimensional, compositional, and sparse, often with thousands of microbial features measured across relatively modest sample sizes. Furthermore, microbiome studies are susceptible to multiple sources of bias that threaten causal validity. Selection bias arises when study populations are not representative of target populations, or when differential loss to follow-up correlates with both microbial exposures and health outcomes (Hernán et al., 2004). Information bias (measurement error) is pervasive due to technical variability in sequencing protocols, batch effects, and DNA extraction methods, which can systematically distort observed microbial abundances and their associations with outcomes (Sinha et al., 2017). Confounding bias is particularly challenging, as factors such as diet, host genetics, medication use, age, geography, and lifestyle can simultaneously influence both the microbiome composition and disease outcomes, creating spurious associations that mimic causal effects (Rupf et al., 2018; Wipperman et al., 2021). Additionally, collider bias can be inadvertently introduced through inappropriate adjustment for intermediate variables or through sample selection based on factors influenced by both exposure and outcome (Griffith et al., 2020). Traditional statistical models frequently struggle under such conditions, particularly when the number of potential confounders exceeds the sample size, which can result in biased estimates, spurious inferences, and poor reproducibility across studies. These issues have contributed to a replication crisis in microbiome science, exemplified by inconsistent findings across large-scale studies, such as the PREDICT cohort (Mazidi et al., 2021), and across disease-specific research in conditions like obesity and osteoarthritis (Kurz et al., 2025). Similarly, mechanistic investigations, such as attempts to link fecal short-chain fatty acids to tumor outcomes, have failed to establish predictive or causal relationships (Sze et al., 2019). These examples underscore the urgent need for methodological frameworks that can disentangle causal signals from confounding, noise, and bias.

Historically, the dominance of correlational machine learning (ML) approaches in microbiome science has contributed to these challenges. While such methods—clustering, classification, and regression-based prediction, have successfully identified candidate microbial biomarkers of disease (Goedert et al., 2015; Zeng et al., 2019), they cannot reliably establish directionality or mechanistic pathways. To address this limitation, the field has increasingly turned to causal machine learning (Causal ML), which integrates modern machine learning with principles from causal inference. This hybrid paradigm incorporates tools such as counterfactual reasoning, instrumental variables (IVs), and structural models to move beyond associations toward actionable causal discovery. Foundational econometric techniques, such as IV estimation (Angrist and Pischke, 2010; Kennedy, 2002; Kitamura and Phillips, 1995), have been adapted into microbiome research through approaches like Mendelian Randomization (MR) (Thomas, 2019). For example, recent studies have leveraged genetic instruments to probe causal microbiome–disease relationships in tuberculosis (Yuan et al., 2024) and asthma (Li R. et al., 2023), highlighting the promise and challenges of adapting econometric strategies to biological data.

More recently, causal ML methods have enabled substantial innovation. Double Machine Learning (Double ML), which employs orthogonalization strategies to isolate treatment effects in the presence of high-dimensional confounding, has been applied to evaluate the effect of interventions such as berberine on cholesterol metabolism (Wu et al., 2022). Directed Acyclic Graphs (DAGs), by encoding prior biological knowledge, allow researchers to identify valid adjustment sets and construct more interpretable causal networks, as demonstrated in Alzheimer's disease–microbiome modeling (Qiu et al., 2024). Mediation analysis frameworks have also been extended with ML to personalize dietary interventions, providing a path toward individualized nutritional therapies informed by causal structures (Ben-Yacov et al., 2023). Parallel advances in time-series causal discovery (Sokolovska et al., 2020) and federated learning platforms such as MiCML (Koh et al., 2025) further illustrate the potential of causal ML to address data fragmentation, privacy concerns, and dynamic causal relationships in microbiome science.

Yet despite these methodological innovations, the application of causal ML in microbiome research remains fragmented and under-standardized. Many studies lack ground-truth validation, cross-cohort replication, or sensitivity analyses, undermining confidence in their conclusions. Without benchmarks, it is difficult to assess the relative merits of different causal ML methods, and without transparent reporting, results remain difficult to reproduce. For instance, while comparative studies have explored the performance of Double ML vs. network analysis (Chen et al., 2025), such efforts remain rare and inconsistent. The absence of standardized reporting frameworks contributes to the opacity of current practice, echoing broader concerns about transparency and reproducibility in computational biology. This is particularly concerning given the high stakes: as microbiome-based therapeutics, from probiotics to fecal microbiota transplantation, move closer to clinical translation, regulatory agencies and clinicians demand rigorous evidence grounded in causal inference.

**Figure 1:**
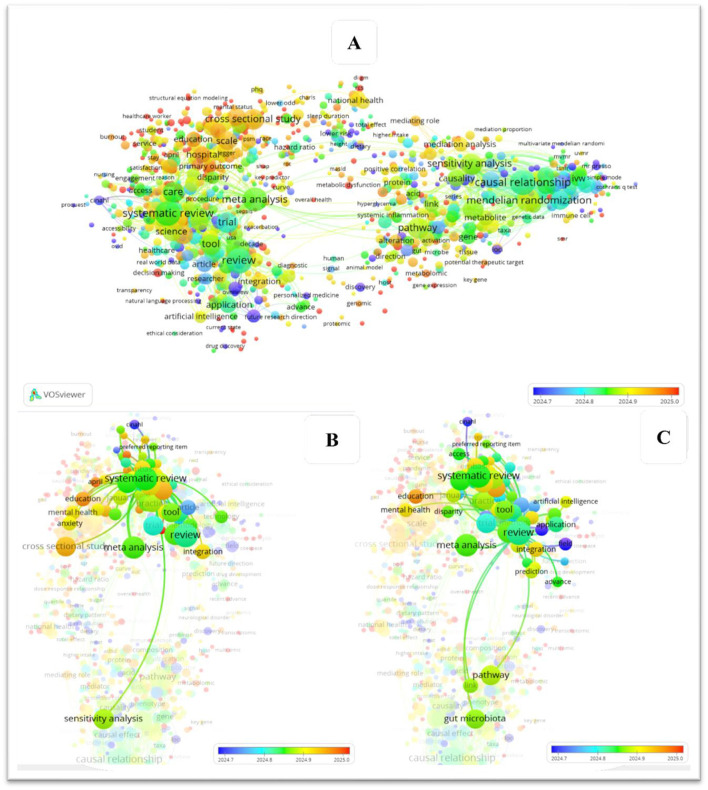
Mapping the evolution of keyword co-occurrence in the literature, with a focus on review studies addressing causal machine learning in health research. The three panels **(A–C)** represent different visualizations of the keyword co-occurrence network and its temporal evolution in studies related to causal machine learning in health research.

**Figure 2:**
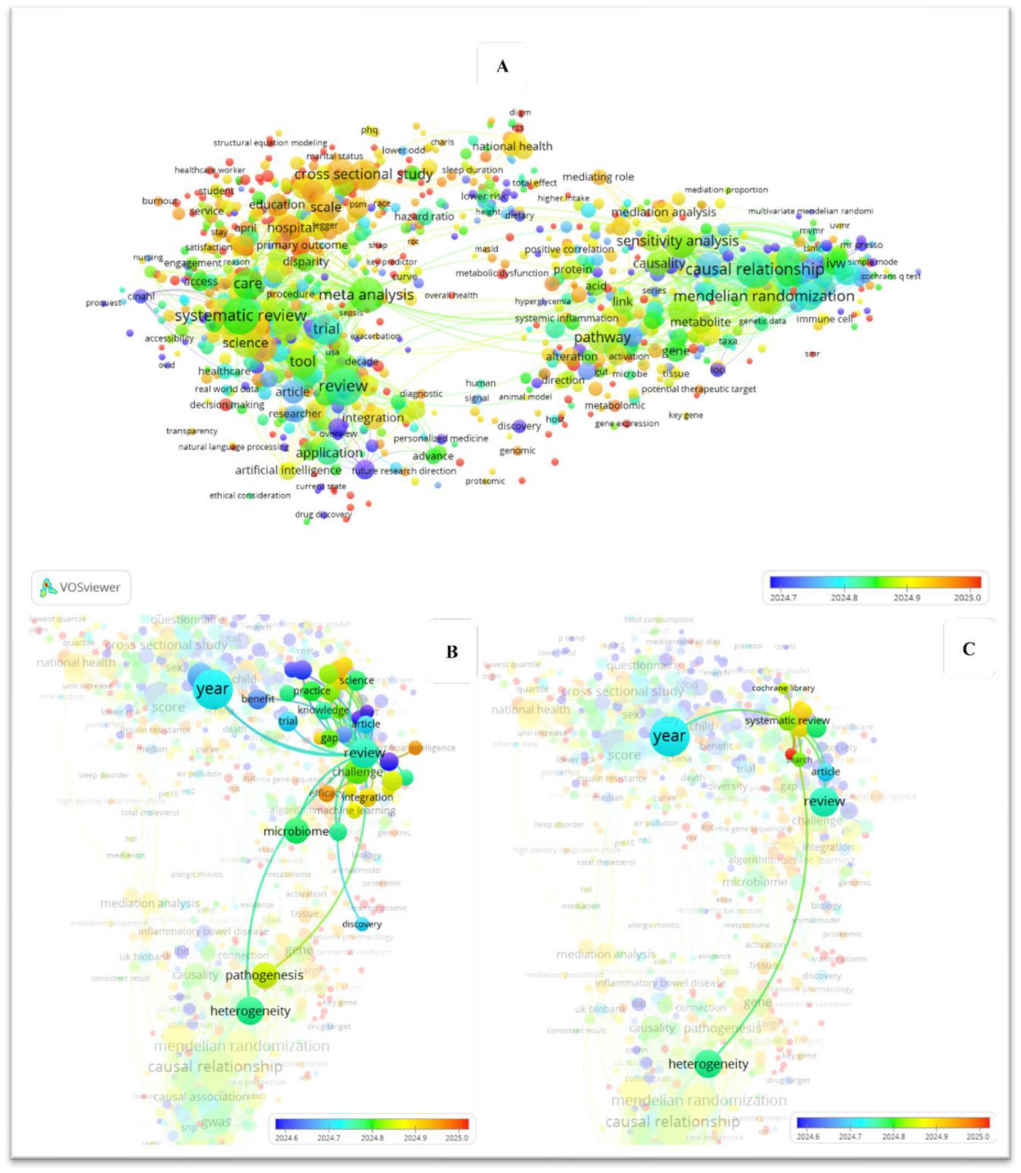
Mapping the evolution of keyword co-occurrence in the literature, with a specific focus on review studies addressing causal machine learning in microbiome research. The three panels **(A–C)** represent different visualizations of the keyword co-occurrence network and its temporal evolution in studies related to causal machine learning in microbiome research.

Although recent reviews have examined causal inference or AI in microbiome science, these efforts remain fragmented and often lack a systematic focus on causal machine learning. Table 1 summarizes existing reviews, their key contributions, and persisting gaps, highlighting how the present work uniquely addresses these limitations. In depth, Figures 1, 2 map the evolution of keyword co-occurrence in the literature, focusing on reviews in causal ML within health and microbiome, as identified through our search in Dimensions.ai. Both figures reveal the absence of a direct link between nodes representing systematic reviews on causal ML and those on the microbiome. The color gradient further shows that this transition has accelerated in recent years (2022–2025), reflecting a surge in microbiome-focused causal inference studies.

**Table 1:** Summary of recent reviews and identified gaps in causal machine learning (Causal ML) for microbiome research.

**Resumed title of existing review (year)**	**Key findings**	**Identified gaps**	**How our systematic review would address this gap**	**References**
Causal machine learning: a survey and open problems (2022/2025)	Comprehensive overview of Causal ML methods across five areas (causal supervised learning, generative modeling, explanations, fairness, reinforcement learning)	General methodological review; does not address microbiome-specific challenges (e.g., compositionality, sparsity, batch effects)	Would bridge the gap between theory and application by systematically evaluating how general Causal ML methods are adapted, validated, and benchmarked for microbiome datasets with unique statistical constraints	Kaddour et al., 2025
Machine learning and AI in the multi-omics approach to gut microbiota (2025)	Explores ML/AI in multi-omics microbiome analysis, including causal inference techniques	Focuses on multi-omics integration but does not systematically assess causal ML for host–microbiome causality or confounder robustness	Would comprehensively categorize and benchmark causal ML approaches (e.g., Double ML, causal forests) for multi-omics microbiome data, testing their robustness against confounding and compositionality challenges	Bajaj et al., 2025
A consensus statement on establishing causality in microbiome research (2025)	Highlights difficulty in establishing causal mechanisms in microbiome-disease relationships	Stresses need for preclinical models and collaboration but lacks focus on computational causal inference for human microbiome data	Would complement experimental strategies by reviewing in silico causal ML frameworks (e.g., IV methods, counterfactual models) that can infer causality from observational human microbiome data, bridging animal–human gaps	Metwaly et al., 2025
Causal inference on microbiome-metabolome relations (2023)	Proposes integrating constraint-based modeling with statistical methods for microbiome-metabolome causal inference	Narrow in scope; does not assess the full spectrum of causal ML (e.g., deep learning, heterogeneous effects)	Would provide a systematic comparison of diverse causal ML methods, including hybrid approaches, for microbiome–metabolome data, evaluating scalability and performance in large datasets	Salim et al., 2023
Mendelian randomization analysis of gut microbiome and Alzheimer's (2025)	Finds initial MR signals between microbes and AD biased by pleiotropy/confounding	Highlights MR limitations (weak instruments, pleiotropy) but does not explore how causal ML could mitigate these	Would review causal ML methods that enhance MR, such as ML-based pleiotropy correction (e.g., MR-Lasso) and hybrid approaches integrating genetic IVs with Double ML	Monaghan et al., 2025
Antibiotic usage as an instrument for causal effect (2025)	Proposes long-term antibiotic use as an IV for microbiome causal inference	Focuses on a single IV type; does not evaluate alternative IVs or broader causal ML approaches	Would catalog and assess all IV-based causal ML strategies (e.g., antibiotic IVs, genetic IVs, natural experiments), testing their validity and generalizability across diseases	Taba et al., 2025
Causal inference in microbiome medicine: principles and applications (2021)	Reviews computational causal inference methods (e.g., MR, mediation) for microbiome medicine	Outdated (2021); omits advances in deep causal ML and cloud-based tool	Would provide an updated synthesis of post-2021 causal ML innovations, including deep causal models, cloud platforms, and large-scale reproducibility frameworks for therapeutic applications	Lv et al., 2021
AI in microbiome analysis and probiotic interventions (2024)	Overview of AI for microbiome analysis (e.g., biomarkers, probiotics)	Emphasizes associative AI/ML; lacks causal inference depth for probiotics or personalized interventions	Would synthesize causal ML methods for optimizing probiotic therapies (e.g., heterogeneous treatment effects, causal forests), advancing personalized microbiome-based interventions	D'Urso and Broccolo, 2024
Recent advances of ML in human gut microbiota study (2023)	Describes transition from observational ML to causal inference in microbiome research	Narrative review; lacks systematic coverage of recent causal ML methods and benchmarks	Would deliver a systematic and comprehensive synthesis of causal ML methods, explicitly covering recent advances, benchmarking standards, and reproducibility guidelines for gut microbiome studies	Tourab et al., 2024
MiCML: a causal ML cloud platform for microbiome medicine (2025)	Introduces MiCML, a cloud-based causal ML platform for microbiome data	Focuses on a single platform; does not compare tools or provide a broader overview of causal ML software	Would evaluate and compare available causal ML platforms (e.g., MiCML, DoWhy, EconML), benchmarking their usability, scalability, and reproducibility for microbiome applications	Koh et al., 2025

Recognizing these gaps, the current review seeks to systematize the rapidly expanding landscape of causal ML applications in microbiome research. We articulate three interrelated objectives. First, we evaluate the spectrum of causal ML methodologies applied to microbiome data, examining their strengths, limitations, and translational potential across real-world datasets. Second, we define and advocate for robust validation frameworks, including synthetic benchmarking, biological plausibility checks, and replication pipelines, to ensure that causal claims are both statistically credible and biologically meaningful. Third, we introduce the STROBE-CML guidelines, an extension of the widely adopted STROBE framework (Cuschieri, 2019; Skrivankova et al., 2021), tailored specifically for causal ML studies in microbiome science, designed to promote transparency, reproducibility, and comparability across studies.

In doing so, we aim to bridge the persistent gap between methodological development and practical implementation. Our synthesis traces the evolution of causal inference in microbiome science, from early mediation analyses (Hu et al., 2024) to recent advances in federated causal ML pipelines (Koh et al., 2025) and novel causal discovery tools (Chen W. et al., 2024). We integrate evidence from computational benchmarks, simulation studies, and empirical applications, constructing a coherent framework for evaluating causal claims. Importantly, we highlight ongoing innovations in reproducibility tools, such as model cards and detailed reporting standards (Hu et al., 2024), which provides a roadmap for transparent dissemination of causal ML findings. Furthermore, by situating causal ML within the broader context of systems biology and multi-omics integration (Miyajima et al., 2024), we illustrate how microbiome research can move toward actionable, mechanistic insights.

The implications of these efforts extend beyond methodological refinement. By providing a decision-support tool that guides researchers in selecting appropriate causal ML methods based on data characteristics, study design, and research objectives, this review seeks to empower investigators to produce more rigorous, reproducible, and clinically translatable findings. Such a framework is essential for building confidence among clinicians, regulators, and patients that microbiome-targeted interventions are grounded in reliable causal evidence. As microbiome science transitions from discovery to intervention, the integration of causal ML with standardized validation and reporting practices represents a critical step toward realizing the promise of microbiome-based diagnostics and therapeutics. By establishing methodological standards and promoting transparency, this review aims to lay the foundation for a new era of reproducible, actionable, and policy-relevant microbiome research (D'Urso and Broccolo, 2024; Lv et al., 2021; Taba et al., 2025).

## 2 Materials and methods

This systematic review was conducted in accordance with the Preferred Reporting Items for Systematic Reviews and Meta-Analyses (PRISMA) 2020 guidelines (Page et al., 2021), with the objective of examining how causal inference methods and machine learning techniques have been jointly applied in human microbiome research with clinical or public health relevance. The search strategy targeted peer-reviewed studies published between January 2015 and May 2025, reflecting the recent surge in computational approaches capable of integrating high-dimensional microbiome data with rigorous causal identification frameworks (Sohrabi et al., 2021).

Two electronic databases were searched: PubMed and Dimensions.ai. This dual-database strategy was selected to capture both traditional biomedical literature (via PubMed's comprehensive MEDLINE indexing) and emerging computational methodologies (via Dimensions.ai's broad interdisciplinary coverage, including preprints and machine learning literature). Our focused approach was deemed appropriate for this emerging interdisciplinary field where relevant studies meeting strict inclusion criteria are limited. Boolean queries were constructed to capture literature at the intersection of microbiome science, causal inference, and machine learning. The microbiome component included terms such as “microbiome,” “gut microbiota,” “gastrointestinal microbiome,” and “16S rRNA.” Causal inference terms encompassed “instrumental variable,” “Mendelian randomization,” “difference-in-differences,” and “causal machine learning,” while the machine learning component included “machine learning,” “random forest,” “deep learning,” “double ML,” “causal forest,” and “Bayesian additive regression trees.” To exclude animal-only or *in vitro* studies, the filter NOT “animal”[tiab] was applied. The final search strings were adapted to the syntax and indexing systems of each database.

The initial search identified 571 records, including 70 from PubMed and 501 from Dimensions.ai. All records were exported in MEDLINE (.nbib) format to preserve structured metadata, including MeSH terms, author affiliations, and structured abstracts. Duplicates were removed using Rayyan.ai's automated detection tool (Ouzzani et al., 2016), followed by manual verification of ambiguous matches, resulting in 477 unique records for title and abstract screening.

Screening was performed in two stages. In the first stage, titles and abstracts were reviewed against four inclusion criteria: studies must (1) focus on the human microbiome; (2) employ a causal inference method; (3) incorporate at least one machine learning approach; and (4) address clinical or public health outcomes. This process yielded 60 records for full-text assessment. In the second stage, full-text articles were reviewed in detail to verify eligibility. Studies were excluded if they used purely causal inference methods without integration of machine learning, applied purely predictive models without causal framing, focused exclusively on animal or *in vitro* models, or were limited to exploratory or correlational analyses. Additional exclusion criteria included non-English language publications and review articles.

A total of 19 studies met all inclusion criteria. These comprised original research articles that explicitly combined causal inference and machine learning within human microbiome contexts, reporting findings relevant to health outcomes. Among these, 15 studies demonstrated particularly strong policy relevance, characterized by discussion of translational applications, public health interventions, or clinical decision-making pathways. These were selected for deeper thematic analysis in the results and discussion sections.

For each included study, a standardized data extraction protocol was applied. Extracted variables included bibliographic information, study design, sample size and population characteristics, type of microbiome data (e.g., 16S rRNA sequencing, metagenomics), causal inference method used, and type of machine learning approach. Where applicable, we documented whether studies addressed potential sources of bias, implemented robustness checks, or discussed limitations in causal interpretation. Special attention was given to identifying whether authors proposed pathways for translating findings into actionable clinical or policy recommendations.

We employed bibliometric and qualitative synthesis software, including VOSviewer (version 1.6.20.0) and Biblioshiny (R-package Bibliometrix), to perform keyword co-occurrence mapping, citation network visualization, and thematic clustering. These tools enabled the identification of research trends, co-authorship networks, and conceptual linkages across studies, providing a structured and reproducible framework for understanding the evolving integration of causal inference and machine learning in microbiome research.

**Figure 3:**
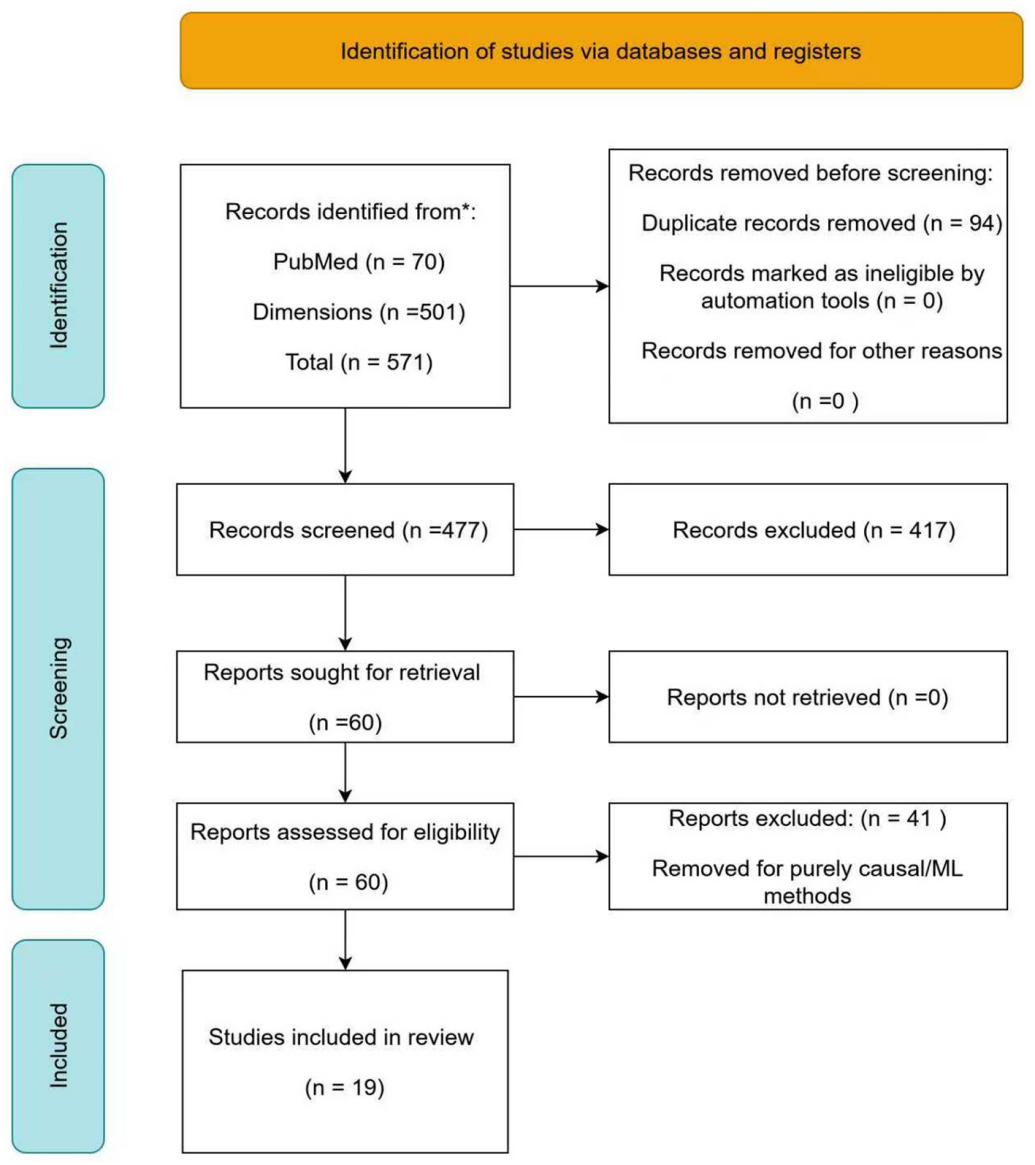
PRISMA flow diagram summarizing the study selection process, from identification to final inclusion in the systematic review methodology.

All steps in the review process were documented to ensure reproducibility. Screening and full-text review were managed entirely within Rayyan.ai, which preserved all metadata in MEDLINE format. The overall selection process is summarized in a PRISMA 2020-compliant flow diagram (Figure 3), illustrating the numbers of records identified, screened, assessed for eligibility, and included, as well as reasons for exclusion at each stage (See Table 2 for the 19 studies and some other relevant ones). This transparent approach ensured methodological rigor and facilitated reproducibility.

**Table 2:** Causal ML in microbiome studies: analyzed papers.

**Number**	**Title**	**Causal-ML methods used**	**Key findings (original, cleaned)**	**References**
1	*Causal analysis between gut microbes, aging indicator, and age-related disease, involving the discovery and validation of biomarkers*	Bidirectional MR; LASSO regression for feature selection	Genetic instruments identified Bacteroides as accelerating aging	Lu et al., 2025
2	*Evidence of a causal and modifiable relationship between kidney function and circulating trimethylamine N-oxide*	Mediation analysis; Explainable ML (XGBoost and SHAP)	Kidney function (eGFR) is a primary modifiable predictor of TMAO; mediation shows gut microbes modulate TMAO–kidney axis	Andrikopoulos et al., 2023
3	*Causal association between gut microbiota and intrahepatic cholestasis of pregnancy*	MR; RF SNP selection	Pregnancy-specific causal taxa identified	Li C. et al., 2023
4	*Dysbiosis signatures of gut microbiota and the progression of type 2 diabetes: a machine learning approach in a Mexican cohort*	SEM; Neural Networks; XGBoost; Random Forest	ML models classified T2D stages; SEM found causal paths from microbiota to insulin resistance	Neri-Rosario et al., 2023
5	*Comparison of initial oral microbiomes of young adults with and without cavitated dentin caries lesions*	Directed acyclic graphs (DAGs); PCA; k-means	DAGs adjusted for confounders (diet, hygiene); detected dysbiosis signatures	Rupf et al., 2018
6	*Association between fecal SCFA and colonic tumor status*	Convergent cross mapping (CCM); RF	CCM and RF found no causal effect of fecal SCFA on colonic tumors	Sze et al., 2019
7	*Gastrointestinal microbiota composition predicts peripheral inflammatory state during treatment of human tuberculosis*	Dynamic causal modeling; LSTM time-series NN	Time-series modeling of host–microbe interactions	Wipperman et al., 2021
8	*Comparative medical ecology of gut microbiomes in NNP disorders*	MR-PRESSO; Federated learning framework	Multi-cohort MR identified disorder-specific causal taxa	Ma et al., 2025
9	*Intestinal flora and inflammatory bowel disease: causal relationships and predictive models*	MR; LASSO; six ML models	MR found 25 causal taxa for IBD; ML models had high predictive ability	Bi et al., 2024
10	*Gut microbiome modulates the effects of a personalized postprandial-targeting diet on cardiometabolic markers*	Causal mediation analysis; ML diet-response prediction	Nine microbial species mediated diet effects on HbA1c and cholesterol	Ben-Yacov et al., 2023
11	*Gut microbiota mediates anti-hypercholesterolemic effect of berberine*	Causal RF	Baseline Alistipes and Blautia predict berberine efficacy	Wu et al., 2022
12	*Gut microbiome features and metabolites in NAFLD*	Microbiome risk score (MRS); RF, SVM, LightGBM	LightGBM performed best; FMT showed causal link between high MRS and NAFLD	Zeng et al., 2024
13	*Gut microbiome signatures during acute infection predict long COVID*	ML predictive models; longitudinal analysis	Acute-phase microbiome predicts Long COVID better than clinical data	Comba et al., 2024
14	*High-dimensional confounding in causal mediation*	Double machine learning; regularized partial correlation network	Tools for high-dimensional confounder selection in gut–brain axis mediation analysis	Chen et al., 2025
15	*MiCML: a causal machine learning platform for microbiome treatment effects*	Causal ML (GRF; RF)	Identifies taxa influencing treatment efficacy (e.g., immunotherapy)	Koh et al., 2025
16	*Childhood obesity risk factors*	Causal discovery (PC-algorithm); multiple imputation and RF	Found indirect causal paths from early exposures to obesity	Foraita et al., 2024
17	*Networks of gut bacteria relate to cardiovascular disease (HELIUS study)*	MR; XGBoost and SHAP	Identified causal cardiovascular-relevant microbes	Warmbrunn et al., 2024
18	*Prenatal lead exposure and gut microbial cliques*	Causal ML pipeline; network analysis	Lead exposure reduces beneficial cliques; links to bilirubin metabolism	Midya et al., 2023
19	*Childhood malnutrition and cognition*	SHAP-interpreted RF; network causality	Malnutrition alters oral microbiota causal link to metabolic shifts with cognitive effects	Portlock et al., 2025
20	*MITRE: microbiome interpretable temporal rule engine*	Bayesian supervised temporal rule learning	Learns interpretable causal-like temporal rules from microbiome time series	Bogart et al., 2019
21	*Causal effects in microbiomes*	Bayesian networks; causal network discovery	Identifies microbial causal factors and quantifies causal effects	Sazal et al., 2021
22	*Unveiling microbial alteration and host gene disarray in Crohn's disease (TAHMC)*	ML-based integrative causal pipeline	Recovered host–microbe interactions; identified diagnostic taxa and genes	Chang et al., 2024
23	*Gut microbiota and acute pancreatitis: MR and nested case–control study*	Bidirectional MR; RF; SVM	Causal microbiota identified for acute pancreatitis	Qu et al., 2024

All any unintroduced acronyms would be introduced more adequately in the next chapter.

## 3 Methodological innovations in causal ML

### 3.1 From regression to causal ML: a conceptual mathematical evolution

At the heart of every causal machine learning method lies a deceptively simple question: *What would happen if we changed one thing, holding all else constant?* This counterfactual query is the essence of causality. Yet answering it requires moving beyond standard statistical models, which are designed to describe associations, not to predict the effects of interventions. In microbiome research, the transition from correlation to causation is hindered by pervasive confounding, high dimensionality, and dynamic host-microbe interactions. Traditional statistical models often fail to distinguish whether microbial shifts are drivers of disease or merely epiphenomena of shared environmental exposures. To address this, causal machine learning rests on two formal frameworks that define causality not in terms of association, but in terms of intervention and counterfactual reasoning: the Potential Outcomes Framework (Huws et al., 2021; Keller and Branson, 2024; Little and Rubin, 2000) and the Structural Causal Model (Pearl, 2009a,b, 2010a,b). These are not competing theories, but complementary languages for expressing causal assumptions and identifying estimable effects.

#### 3.1.1 The potential outcomes framework: defining causality via counterfactuals

At the heart of causal inference lies a simple but profound idea: to understand the effect of a microbial exposure, such as the abundance of *Faecalibacterium prausnitzii* (*F. prausnitzii*), on a host outcome like inflammation, we must ask: What would have happened to the same individual if their microbial profile had been different? This counterfactual question is formalized in the potential outcomes' framework (Huws et al., 2021; Keller and Branson, 2024; Little and Rubin, 2000). For each individual *i*, we define:

- *Yi*(1) : the outcome [e.g., C-reactive protein (CRP) level] if individual *i* had high *F. prausnitzii*,
- *Yi*(0) : the outcome if individual *i* had low levels.

The individual causal effect is:


$$\begin{array}{l} \tau_{i}=Y_{i}\left(1\right)-Y_{i}\left(0\right) \end{array}$$

But we can never observe both outcomes simultaneously—one is always missing (the counterfactual). Therefore, causal inference focuses on estimating the Average Treatment Effect (ATE) across a population:


$$\begin{array}{l} ATE=E\left[Y\left(1\right)-Y\left(0\right)\right] \end{array}$$

This quantity can be identified from observational data under two key assumptions:

- Ignorability: all confounders are measured and can be adjusted for,
- Overlap: every individual has a non-zero probability of receiving either exposure level.

These assumptions are critical, and often violated in microbiome studies due to unmeasured lifestyle or genetic factors.

#### 3.1.2 Bias structures and systemic methodological challenges in microbiome causal inference

Before proceeding with advanced estimation methods, it is essential to formally characterize the sources of bias that threaten causal inference in microbiome research. Two analytically distinct but closely related layers of concern arise in this literature. The first involves traditional epidemiological bias, which directly threatens internal validity through violations of causal identification assumptions. The second involves broader systemic methodological issues, including limited cross-cohort validation, model overfitting in high-dimensional settings, selective reporting, and limited analytical transparency. These systemic issues are not bias parameters within the potential outcome's framework; rather, they create conditions under which classical biases are more likely to arise, persist, or remain undetected. A rigorous framework must therefore distinguish these layers while recognizing their interaction (Dickerman and Hernán, 2020; Ioannidis, 2019; Pearl, 2009a, 2010b).

We distinguish four primary bias structures within the causal inference framework.

Confounding bias occurs when a common cause of both the microbial exposure and the outcome is not adequately controlled. In the potential outcome's framework, this violates the ignorability assumption: *Y*(*x*) *not*⊨*X*.

Where the potential outcome under exposure level x, Y(x), is not independent of the observed exposure X. For example, antibiotic use may influence both gut microbial diversity and subsequent infection risk, thereby confounding the apparent protective effect of high diversity (Dror et al., 2017; Griffith et al., 2020). In directed acyclic graph (DAG) notation, confounding induces a backdoor path between exposure and outcome that must be blocked through appropriate adjustment or instrumental variables. Given the dense correlation structure of microbiome features and their shared determinants (diet, medication, and environment), residual confounding remains a central identification challenge.

Selection bias arises when inclusion into the study sample—or retention within it—is related to both exposure and outcome. This includes differential participation (e.g., healthier individuals more likely to provide stool samples), differential attrition (e.g., sicker patients dropping out of longitudinal studies), and conditioning on post-exposure variables affected by both exposure and outcome (Goren et al., 2021; Griffith et al., 2020). While collider bias is often considered a subtype of selection bias, it can also arise during analytic conditioning rather than through sampling alone. Selection bias generally cannot be resolved through standard covariate adjustment and instead requires careful design, inverse probability weighting, or formal sensitivity analyses.

Information bias occurs when microbial exposures or outcomes are measured with error. Non-differential measurement error (random with respect to outcome status) typically attenuates effect estimates toward the null, whereas differential measurement error (systematically related to outcome status) may bias estimates in either direction (Keogh and White, 2014). In microbiome research, batch effects, DNA extraction protocols, sequencing depth variability, and bioinformatic preprocessing pipelines are major sources of measurement distortion (Sinha et al., 2017). Without appropriate technical correction prior to causal modeling, estimated effects may reflect laboratory artifacts rather than biological mechanisms.

Collider bias is induced when analyses condition on a variable that is causally influenced by both the exposure and the outcome, or by causes of each. Conditioning on such a variable opens a non-causal pathway and can generate spurious associations even in the absence of a true causal effect (Pearl, 2009a, 2010b). For example, adjusting for inflammatory markers when studying the microbiome–disease relationship may introduce bias if inflammation is affected by both microbiome composition and disease progression. In high-dimensional settings, data-driven variable selection procedures may inadvertently condition on colliders, thereby distorting causal interpretation.

Beyond these classical epidemiological biases, microbiome research faces broader systemic methodological challenges. High dimensionality relative to sample size increases the risk of overfitting, post-selection bias, and regularization-induced distortion. Limited cross-cohort validation undermines transportability of findings. Selective reporting of statistically significant taxa and opaque analytical pipelines weaken reproducibility. While these weaknesses do not correspond to violations of ignorability or consistency assumptions directly, they affect the credibility, robustness, and external validity of estimated effects.

Conceptually, traditional epidemiological biases threaten internal validity, meaning the unbiased identification of causal effects within the study population. Systemic methodological weaknesses primarily threaten robustness and transportability, that is, whether estimated effects generalize beyond the original sample and remain stable under alternative analytical specifications. Importantly, these domains interact: overfitting may conceal residual confounding, inadequate reporting may obscure measurement error, and lack of validation may mask instability in causal estimates.

A coherent causal framework for microbiome research must therefore address both identification-level bias structures and systemic methodological rigor simultaneously. Only by integrating formal causal assumptions with transparent, validated, and reproducible computational workflows can microbiome studies move from associative pattern detection toward credible causal inference.

#### 3.1.3 The structural causal model: causality as intervention

An equivalent but graphically intuitive framework is the Structural Causal Model (SCM), introduced by Pearl (2009a, 2010a,b). In an SCM, variables are linked by functional relationships:


$$\begin{array}{l} Y=f\left(X,U\right) \end{array}$$

where: *Y* is the outcome, *X* is the exposure (e.g., microbial taxon), *U* represents unobserved factors (e.g., host genetics, early-life environment), *f*(·) is a structural function that may be linear or non-linear.

Causality is defined through the do-operator:


$$\begin{array}{l} Causal\;Effect=Y|do\left(X=x\right)-Y|do\left(X=x^{\prime }\right) \end{array}$$

Here, *do*(*X* = *x*) represents an intervention, setting *X to x*, as opposed to merely observing *X* = *x or X* = *x*′. This distinction is essential: observing high *F. prausnitzii* in healthy individuals does not imply that increasing it will improve health; only an intervention can reveal the true effect.

SCMs are often visualized using Directed Acyclic Graphs (DAGs), which make assumptions about confounding, mediation, and colliders explicit. For example, a DAG might encode that diet influences both microbiota and inflammation, requiring adjustment to avoid biased estimates.

#### 3.1.4 Bridging to causal machine learning: high-dimensional adjustment via orthogonal estimation

While these frameworks define causality, they do not solve the practical problem of high-dimensional confounding, a hallmark of microbiome data. Thousands of microbial features, clinical covariates, and technical variables may all act as confounders, overwhelming traditional adjustment methods. The breakthrough came with the realization that if we could separate the estimation of the causal effect from the modeling of confounders, we could use flexible machine learning algorithms (e.g., random forests, neural networks) to model nuisance parameters without biasing the final estimate. This led to orthogonal or doubly robust estimators (Chernozhukov et al., 2018; Funk et al., 2011; Oprescu et al., 2019b), such as:


$$\begin{array}{r} \hat{\tau }=\frac{1}{n}\sum_{i=1}^{n}[{\hat{\mu }}_{Y}\left(1,W_{i}\right)-{\hat{\mu }}_{Y}\left(0,W_{i}\right)+\frac{X_{i}}{\hat{e}\left(W_{i}\right)}\left(Y_{i}-{\hat{\mu }}_{Y}\left(1,W_{i}\right)\right)\\ +\;\frac{1-X_{i}}{1-\hat{e}\left(W_{i}\right)}\left(Y_{i}-{\hat{\mu }}_{Y}\left(0,W_{i}\right)\right)\;] \end{array}$$

Where: *W* is a vector of confounders [e.g., age, diet, Body Mass Index (BMI)], ${\hat{\mu }}_{Y}\left(1,W_{i}\right),\;{\hat{\mu }}_{Y}\left(0,W_{i}\right)$: ML-based predictions of the outcome under treatment and under control, and outcome, ê(*W*): estimated propensity score. The residuals remove confounding influence, isolating the causal signal.

This structure, residualizing both treatment and outcome, is the mathematical core of modern causal ML. It enables robust inference even when the number of confounders is large, provided cross-fitting is used to prevent overfitting.

We can also express this evolution as:


$$\begin{array}{l} Y=f_{\theta }\left(X,Z\right)+\varepsilon \end{array}$$

where *f*_θ_ is learned by ML, and *Z* represents observed confounders. This bridges the SCM to modern methods like Double ML, Deep IV, and Causal Forests.

### 3.2 A unified taxonomy of causal ML methods in microbiome research

The mathematical foundation established in Section 3.1, centered on counterfactual reasoning, orthogonal estimation, and double robustness, has given rise to a rich and rapidly expanding ecosystem of causal machine learning methods. These approaches differ in implementation, computational complexity, and data requirements. Nevertheless, they share a unifying principle: the separation of causal effect estimation from the modeling of nuisance parameters, such as high-dimensional confounders or complex outcome processes. This decoupling enables the integration of flexible machine learning algorithms, ranging from random forests to neural networks, without sacrificing statistical rigor. Thus, making it possible to draw credible causal inferences from observational microbiome data.

However, the growing diversity of available methods presents a significant challenge for researchers: how to select the most appropriate tool for a given biological question, data structure, and computational context. To address this, we present Table 3:

**Table 3:** Taxonomy of causal machine learning methods in microbiome research, categorized by approach, with key assumptions, strengths, limitations, and example applications.

**Method**	**Core assumptions**	**Data type handled**	**Key strengths**	**Limitations and challenges**	**Example microbiome application**	**Reference of the example and other useful references**
**Adjustment-based estimators**						
Double/Debiased ML (DML)	Ignorability, overlap, no unmeasured confounding	Cross-sectional, high-dimensional	Doubly robust; handles ML nuisance models	Requires large N; sensitive to model misspecification	Estimating the causal effect of an environmental exposure (e.g., diet, medication) on preterm birth risk, accounting for confounding by the gut microbiome.	Chen M. et al., 2024; Chen et al., 2025; Chernozhukov et al., 2018; Fuhr et al., 2024; Miao et al., 2025
Orthogonal random forest (ORF)	Same as DML; stable trees	Heterogeneous populations	Provides inference for individual-level effects	Computationally heavy; needs large samples	Identifying subgroups responding to probiotics by baseline microbiota	Oprescu et al., 2019a,b
Targeted maximum likelihood estimation (TMLE)	Ignorability, positivity	Small-sample observational	Doubly robust, efficient; works in small cohorts	Sensitive to extreme propensity scores	Breastfeeding duration → infant microbiome diversity	Luque-Fernandez et al., 2018; Schnitzer et al., 2014; Smith et al., 2025
G-computation (G-formula)	Correct outcome model, no unmeasured confounding	Time-varying exposures	Flexible for dynamic interventions	Highly sensitive to model misspecification	Decomposing the total effect of an exposure (e.g., antibiotic use) on a health outcome into direct effects and indirect effects mediated through the microbiome	Hiu et al., 2025; Rogawski et al., 2016; Zhang et al., 2023
Inverse probability weighting (IPTW)	Correct PS model, positivity	Cross-sectional, binary exposures	Simple, intuitive; widely understood	Unstable if PS near 0/1; not doubly robust	Comparing survival outcomes (e.g., disease remission, infection risk) between patient groups with different microbiome profiles (e.g., high vs. low diversity) by balancing confounders	Cook et al., 2020; Takada et al., 2021
High-dimensional propensity score (hdPS) and ML	Measured confounders, positivity	EHR-linked microbiome data	Automates confounder selection	May miss unrecorded variables	A methodological framework for addressing technical and non-causal confounding (e.g., batch effects) in high-dimensional microbiome epidemiology studies	Karim, 2025; Ponsonby, 2021; Yount et al., 2020
**Instrumental variable (IV) methods**						
Two-stage least squares (2SLS-ML)	Valid instrument, exclusion restriction	Genetic or environmental instruments	Reduces bias from unmeasured confounding	Weak instruments → bias	SNP → microbial pathway → metabolite association	Ailer et al., 2025; Namkung, 2020; Taba et al., 2025
DeepIV (Neural Instrumental Variables)	Same as IV; no unmeasured confounding	Non-linear exposure-response	Captures complex, non-linear dose-response	Needs strong instruments; hard to validate	Infer causal relationships between microbial features and host phenotypes (e.g., disease outcomes, metabolic responses)	Hernández Medina et al., 2022; Przymus et al., 2025
Mendelian Randomization (MR/MR-LASSO)	No pleiotropy, instrument strength	GWAS + microbiome (MWAS)	Mimics RCT; growing data availability	Horizontal pleiotropy; weak instruments	Estimates the causal relationship between both oral microbes and cancer	Wu et al., 2025
**Structure learning and graphical models**						
Directed acyclic graphs (DAGs)	Causal sufficiency, no feedback	Any (used in design phase)	Prevents collider/over adjustment bias	Subjective; requires domain expertise	Address challenges like weak instruments and pleiotropy in high-dimensional genetic data	Burgess et al., 2020
NOTEARS (DAG Learning)	Linear/additive noise; acyclicity	Cross-sectional, moderate N	Data-driven structure discovery	Assumes no unmeasured confounders	Used to infer directed microbial interactions, host-microbe causal pathways, and functional mechanisms	Sun et al., 2023; Zheng et al., 2018
Invariant causal prediction (ICP)	Invariant mechanism across environments	Multi-environment data (e.g., cohorts)	High precision in causal discovery	Requires ≥2 environments	Identifies the set of microbial features (e.g., taxa, genes) whose relationship with a host phenotype (e.g., BMI, inflammation) is constant across all these environments	Li and Goeman, 2024; Mey and Castro, 2024; Pfister et al., 2019
**Longitudinal and dynamic models**						
Granger causality (VAR)	Stationarity, no unmeasured confounders	Dense time series	Simple, interpretable	Fails with feedback loops	Identifies directed taxon-taxon relationships (e.g., Bacteroides on Prevotella) in gut microbiomes, distinguishing mutualism, competition, or parasitism	Cai et al., 2022; Mainali et al., 2019
Convergent cross-mapping (CCM)	Underlying dynamical system	Non-linear, deterministic systems	Detects causality in closed loops	Needs very dense sampling	Using time-lagged vectors of microbial abundance or host phenotype data to reconstruct high-dimensional dynamics	Maher and Hernandez, 2015; Sun et al., 2024
Dynamic Causal Bayesian Networks (DCBNs)	Markov property, faithfulness	Multi-omic time series	Handles feedback, time-lagged effects	Computationally intensive	DCBNs use DAGs to model dependencies between variables (e.g., microbial taxa, metabolites, host genes) within and across time points, ensuring acyclicity to avoid logical loops	Hammond and Smith, 2025; McGeachie et al., 2016
**Scalable an federated methods**						
Federated double ML	Homogeneous causal effect across sites	Distributed datasets (multi-center)	Privacy-preserving; scalable	Assumes transportability across cohorts	Estimating the effect of microbial taxa (e.g., *Akkermansia muciniphila*) on host phenotypes (e.g., insulin resistance) while adjusting for confounders (diet, genetics) across diverse populations	Khellaf et al., 2025; Rémi et al., 2025
Invariant risk minimization (IRM)	Invariant mechanism across environments	Multi-cohort, heterogeneous data	Generalizable, reproducible predictors	May discard predictive (non-causal) signals	IRM can identify microbial features (e.g., *Faecalibacterium prausnitzii*) whose effects on neuroinflammation are consistent across cohorts, despite technical heterogeneities	Montoro et al., 2022; Zhu et al., 2020
**Synthetic and quasi-experimental designs**						
Synthetic control	No unmeasured confounders; stable pre-trend	Single intervention unit	Creates counterfactual control	Hard to generalize	Estimate causal effects of interventions (e.g., policies, treatments) on a single unit (e.g., individual, region) by constructing a weighted combination of control units that mimic the treated unit's pre-intervention outcomes and covariates	Krajewski and Hudgens, 2024; Rehkopf and Basu, 2018; Krajewski and Hudgens, 2024; Rehkopf and Basu, 2018
Synthetic difference-in-differences (SDID)	Parallel trends, no anticipation	Few treated units	More robust than DiD	Rarely used in microbiome	Creating synthetic controls: for a cohort receiving an intervention [e.g., fecal microbiota transplantation (FMT)], SDID can construct a synthetic control from multiple untreated cohorts (e.g., individuals with similar baseline microbiome profiles)	Bolsega et al., 2021; Clarke et al., 2023

Taxonomy of Causal Machine Learning Methods in Microbiome Research, a comprehensive synthesis of 18 methods grouped into six functional categories. Table 3 is not merely a summary; it is a decision-enabling tool. It compares each method along critical dimensions: core assumptions, data compatibility, strengths, limitations, real-world microbiome applications, and foundational references. By organizing methods thematically and highlighting their underlying causal logic, the table allows researchers to move beyond algorithmic novelty and instead select methods based on biological plausibility, study design, and reproducibility.

This taxonomy illustrates how a single causal foundation, the potential outcomes framework enhanced by machine learning, has been adapted to meet the heterogeneous demands of microbiome research. From estimating the effect of a single taxon in a cross-sectional cohort to reconstructing dynamic microbial networks over time, or validating findings across international biobanks, each method extends the core causal structure to address specific analytical challenges.

#### 3.2.1 Adjustment-based estimators: controlling for high-dimensional confounding

These methods are designed to estimate average or heterogeneous treatment effects while adjusting for observed confounders, even when their number is large relative to sample size. Double/Debiased Machine Learning (DML) stands out as one of the most influential advances in recent years. Rooted in the Neyman-orthogonal estimation framework, DML decouples the estimation of causal effects from nuisance parameters, such as the outcome regression and propensity score, through cross-fitting and residualization. This orthogonalization allows for the use of flexible, data-adaptive ML models (e.g., gradient boosting, neural networks) to control for high-dimensional confounders without compromising inference validity. In microbiome applications, DML has been deployed to estimate the causal effect of individual taxa, such as *Akkermansia muciniphila*, on host outcomes like insulin resistance, adjusting for dozens of covariates including diet, BMI, and medication history (Davis and Heller, 2017). Its double robustness ensures consistency even if one of the nuisance models is misspecified, enhancing resilience against model error, a common pitfall in microbiome analyses.

Targeted Maximum Likelihood Estimation (TMLE) offers similar robustness with superior efficiency in smaller datasets. A study on infant gut microbiota used TMLE to estimate the causal effect of breastfeeding duration on microbial diversity, adjusting for socioeconomic and delivery-mode confounders (Völzke et al., 2022). TMLE's targeted updating procedure ensures the final estimate is optimized for the causal parameter of interest, rather than overall model fit.

For settings where confounders are numerous but poorly specified, High-Dimensional Propensity Score (hdPS) with ML automates variable selection from electronic health records, reducing bias due to unmeasured clinical factors (Steen, 2021). When interest lies in individual-level heterogeneity, such as differential responses to probiotics across genotypes, Orthogonal Random Forest (ORF) and Causal Forests enable discovery of effect modifiers, though they require sufficient sample size to ensure stable inference (Athey et al., 2019; Athey and Imbens, 2019; Athey and Wager, 2019; Oprescu et al., 2019b).

#### 3.2.2 Instrumental variable methods: addressing unmeasured confounding

When key confounders are unmeasured or unknown, instrumental variable (IV) approaches offer a pathway to causal identification by leveraging exogenous sources of variation. Two-Stage Least Squares (2SLS-ML) uses predictors, such as genetic variants or birth mode, that influence the microbial exposure but not the outcome directly, enabling estimation of causal effects under the exclusion restriction assumption. This approach has been widely adopted in Mendelian Randomization (MR) studies, where SNPs serve as instruments to test whether microbial taxa causally influence disease risk (Wu et al., 2025). For instance, SNPs associated with lactase persistence have been used to probe the causal impact of *Bifidobacterium* spp. levels on bone mineral density, leveraging the known link between lactose digestion and bifidobacterial growth.

To improve instrument selection in high-dimensional genetic data, MR-LASSO combines penalized regression with MR, enhancing power and specificity (Chen G. et al., 2024; Groot et al., 2020; Monti et al., 2024; Wu et al., 2024; Xue et al., 2021). For modeling complex, non-linear exposure-response relationships inherent to host-microbiome interactions—such as the threshold effects of a keystone taxon or the saturating dose-response of a probiotic—advanced causal machine learning methods are required. The DeepIV framework (Hartford et al., 2017) is particularly powerful in this context, as it employs deep neural networks within an instrumental variable (IV) setup to model intricate, non-uniform biological dose-response curves, even in the presence of unobserved confounding. This capability makes it uniquely suited for microbiome studies where relationships are rarely linear. For instance, its application could be extended to model the causal effect of microbial abundance on a host phenotype, not as a simple linear effect, but as a complex function that may have critical thresholds or diminishing returns. This aligns with the growing emphasis on precision and biomarker discovery in biological dose-response modeling, where capturing this complexity is essential for generating clinically actionable insights (Gutierrez et al., 2024).

#### 3.2.3 Structure learning and graphical models: from association to causal architecture

Beyond estimating effects, researchers increasingly seek to discover the underlying causal architecture of microbiome-host systems. Directed Acyclic Graphs (DAGs) provide a formal language for encoding causal assumptions and guiding analytical decisions. DAGs represent variables as nodes and causal relationships as directed edges, enabling researchers to visualize and test the implications of different causal structures. In microbiome research, DAGs have been instrumental in clarifying the role of mediators (e.g., short-chain fatty acids in gut-brain axis signaling), identifying backdoor paths through which confounding operates, and determining minimal sufficient adjustment sets. For example, a DAG might encode the hypothesis that antibiotic exposure influences cognitive decline via its effect on gut microbial diversity, with inflammation serving as a mediator. Such a structure allows for the identification of estimands like natural direct and indirect effects using mediation analysis frameworks (Gupta et al., 2021). Importantly, DAGs do not replace statistical analysis but rather precede it, ensuring that modeling choices are aligned with biological plausibility and reducing the risk of overadjustment or collider bias.

Data-driven alternatives like Non-combinatorial Optimization via Trace Exponential and Augmented Lagrangian for Structure Learning (NOTEARS) learn DAG structures directly from data using continuous optimization, enabling discovery of microbial interaction networks without combinatorial search (Sun et al., 2023; Zheng et al., 2018). Complementing this, Invariant Causal Prediction (ICP) identifies predictors whose effect remains stable across environments (e.g., cohorts or populations), offering a powerful strategy for distinguishing causal drivers from spurious associations (Li and Goeman, 2024; Mey and Castro, 2024; Pfister et al., 2019).

#### 3.2.4 Longitudinal and dynamic models: capturing temporal dynamics

Given the dynamic nature of microbial communities, temporality is a key criterion for causality. Static cross-sectional analyses are inherently limited in their ability to establish whether microbial shifts precede or follow host changes. Granger causality, particularly in its regularized form (e.g., GLASSO), tests whether past values of one microbial taxon predict future values of another or a host marker. It has been used to show that *Bacteroides* fluctuations consistently precede changes in secondary bile acid metabolism, supporting a regulatory role in host metabolism (Mainali et al., 2019). More recently, convergent cross-mapping (CCM) and transfer entropy have gained traction for detecting causal interactions in non-linear, deterministic systems, offering advantages in ecological contexts where feedback loops and bistability are common (Sugihara et al., 2012).

Dynamic Causal Bayesian Networks (DCBNs) are powerful for modeling time-lagged dependencies and feedback loops among multiple variables in longitudinal studies. This makes them ideal for studying chronic diseases like Inflammatory Bowel Disease (IBD), where they can be used to predict impending clinical flares from preceding microbial community volatility and trajectories. The application of such advanced time-series causal models is a growing frontier in microbiome research for uncovering host-microbe dynamics (Nitschke et al., 2024).

#### 3.2.5 Scalable and federated inference: enabling multi-center validation

As microbiome research becomes increasingly collaborative, methods that support privacy-preserving, cross-institutional analysis are essential. Federated causal learning enables the estimation of causal effects across decentralized datasets without sharing raw individual-level data. Federated learning architectures allow local models to be trained on site-specific data, with only model updates (e.g., gradients or parameter estimates) shared centrally for aggregation. When combined with causal ML principles, such as inverse probability weighting or doubly robust estimators, this framework supports cross-cohort causal inference while preserving participant confidentiality. For example, a recent study applied federated Double ML to harmonize data from three independent cohorts investigating the causal effect of *Akkermansia muciniphila* abundance on metabolic syndrome, achieving consistent effect estimates despite differences in sequencing protocols and population demographics (Koh et al., 2025). This represents a significant step toward large-scale, generalizable causal claims that transcend single-center biases.

Invariant Risk Minimization (IRM) complements this by learning representations that generalize across environments, filtering out population-specific noise to yield more reproducible microbial signatures. Previous research introduced the causal representation learning framework, Invariant Risk Minimization (IRM), which is particularly valuable in global microbiome studies where environmental heterogeneity threatens reproducibility. By identifying features with stable causal relationships to the outcome, IRM provides a powerful approach for deriving generalizable biomarkers from multi-cohort data. This has been demonstrated in emerging applications to microbial ecology and host–disease prediction (Arjovsky et al., 2020).

#### 3.2.6 Synthetic and quasi-experimental designs: estimating interventions from observational data

Finally, for settings where randomized trials are infeasible, quasi-experimental methods mimic intervention designs. Synthetic control constructs a counterfactual control group from weighted combinations of non-intervention units, useful for evaluating the impact of events like fecal microbiota transplantation (FMT) in single-patient or single-cohort studies. Synthetic Difference-in-Differences (SDID) extends this with improved weighting and robustness, offering a modern alternative to classical DiD in settings with few treated units (Clarke et al., 2023; Dench et al., 2024; Jones-Smith et al., 2024).

#### 3.2.7 Integration across molecular layers: multi-omic and latent causal discovery

Beyond methodological categories, recent advances focus on biological integration and systems-level inference. Multi-omic causal integration enhances the biological interpretability of findings by combining metagenomic, metatranscriptomic, metabolomic, and host transcriptomic data within a unified causal framework (Bing et al., 2022; Chetty and Blekhman, 2024; Mohammadi-Shemirani et al., 2023; Santiago-Rodriguez and Hollister, 2021). Structural equation models (SEMs) and Bayesian networks have been adapted to model layered interactions, allowing for the identification of key mediators and effect modifiers. For example, a study integrating shotgun metagenomics and plasma metabolomics used a causal mediation model to demonstrate that the effect of *Faecalibacterium prausnitzii* on reduced systemic inflammation was largely mediated by increased production of butyrate, a short-chain fatty acid with known anti-inflammatory properties (Liu et al., 2020). Such integrative approaches not only strengthen causal claims but also generate testable hypotheses for experimental validation.

Finally, causal representation learning is an emerging frontier that seeks to discover latent causal variables from high-dimensional microbiome data. Rather than treating individual taxa as atomic units, these methods aim to identify functionally coherent microbial modules or “causal factors” that operate as unified drivers of host outcomes. Autoencoders, variational inference, and invariant risk minimization have been combined with causal constraints to learn representations that are stable across environments and predictive of interventional effects. This paradigm holds promise for moving beyond taxonomic-centric analyses toward a systems-level understanding of microbiome function.

Together, these methods form a coherent causal ecosystem, rooted in a shared mathematical foundation but diversified to meet the multifaceted challenges of microbiome science. Their existence in parallel, not competition, reflects the complexity of host-microbe systems, where no single method can address all questions. The choice among them must therefore be guided not by algorithmic elegance, but by biological plausibility, data structure, and practical constraints. A principle that underpins the decision support tool introduced in Section 6.

## 4 Validation frameworks for robust causal inference

### 4.1 Benchmarking standards

As causal ML methods proliferate, so too does the need for standardized, rigorous evaluation frameworks that explicitly address both traditional epidemiological biases and modern computational challenges. Without reliable benchmarks, it is difficult to compare methods, assess their performance under realistic conditions, detect overfitting and false positives, or evaluate robustness to violations of key assumptions such as no unmeasured confounding or correct model specification. Validation frameworks serve a dual purpose: (1) assessing method performance under controlled conditions where ground truth is known, and (2) evaluating the extent to which methods mitigate specific sources of bias [confounding, selection, measurement error, and collider bias] that threaten causal validity in observational microbiome studies (Hernán and Robins, 2025). Two complementary benchmarking strategies have emerged as essential components of robust validation: synthetic data generation for controlled evaluation of bias reduction under known data-generating processes, and biological plausibility checks for assessing whether inferred causal relationships are consistent with mechanistic knowledge and experimental evidence. Together, these strategies provide a comprehensive framework for distinguishing genuine causal signals from artifacts of bias, overfitting, or model misspecification.

Synthetic data generation offers a powerful solution to the absence of ground-truth causal relationships in real-world microbiome datasets. By simulating microbial communities with known interaction networks, compositional constraints, and host response mechanisms, researchers can evaluate the accuracy, precision, and calibration of causal ML algorithms in controlled settings while systematically introducing specific bias structures to test method robustness. For example, synthetic benchmarks can simulate unmeasured confounding (violating ignorability), differential measurement error in taxonomic classification (information bias), or study designs with non-random sampling (selection bias) to assess whether causal ML methods produce unbiased effect estimates or appropriately flag violations of their core assumptions (Keogh and White, 2014; Wang et al., 2023). This controlled evaluation is critical because real microbiome datasets rarely provide the counterfactual data needed to validate causal claims directly.

Recent advances have enabled the creation of synthetic microbiomes that closely mimic real data in terms of sparsity, overdispersion, and phylogenetic structure. For example, the microbiome giBenchmark package generates count data using log-normal multinomial models with predefined correlation structures and causal pathways, allowing users to introduce confounding, mediation, and non-linear effects systematically (Wang et al., 2023). In one benchmarking study, ten causal ML methods were tested on synthetic datasets with known treatment effects; only Double ML and targeted maximum likelihood estimation (TMLE) consistently recovered unbiased estimates across varying sample sizes and confounder dimensions, highlighting the importance of method selection (Chernozhukov et al., 2018; Luque-Fernandez et al., 2018; Smith et al., 2025; Wang et al., 2023). Such efforts provide much-needed empirical evidence for method performance and help identify conditions under which certain approaches fail.

However, synthetic benchmarks alone are insufficient. They rely on modeling assumptions that may not reflect biological reality, such as simplified interaction topologies or linear dose-response relationships. Therefore, biological plausibility checks must accompany computational validation to ensure that inferred causal relationships are consistent with existing knowledge and amenable to experimental interrogation. One effective strategy is multi-omic integration, where causal inferences from 16S rRNA or metagenomic data are cross-validated against metabolomic or proteomic profiles. For instance, if a causal model identifies *Clostridium scindens* as a driver of reduced atherosclerosis risk, one would expect to observe corresponding changes in secondary bile acid metabolism, given this species' known role in converting primary to secondary bile acids. Discrepancies between predicted and observed molecular signatures raise red flags about the validity of the causal claim (Bucci et al., 2023).

Additionally, experimental validation remains the gold standard for confirming causality (Koch and Röhmel, 2004; Meinshausen et al., 2016). These examples underscore the necessity of closing the loop between computational prediction and biological experimentation.

### 4.2 Reproducibility pipelines

Beyond benchmarking, long-term reproducibility depends on systematic pipelines that promote transparency, consistency, and external validation. Cross-study validation is arguably the most stringent test of a causal claim's generalizability. A finding that replicates across independent cohorts, differing in geography, ethnicity, sequencing platform, or clinical protocol, is far more credible than one observed in a single dataset. Yet, such replication remains rare in microbiome research. A recent review found that fewer than 15% of reported microbial associations were validated in more than one cohort, let alone under causal frameworks (Rodriguez et al., 2024). To address this, several consortia, including the Integrative Human Microbiome Project (iHMP) and the Microbiome Quality Control Project (MBQC), have begun promoting standardized protocols and data sharing to facilitate cross-cohort causal analysis. One successful example involved the replication of a causal effect of *Prevotella copri* on rheumatoid arthritis risk across three geographically distinct cohorts using harmonized taxonomic profiling and consistent adjustment for confounders, demonstrating the feasibility and value of multi-center causal inference.

Equally important are sensitivity analyses, which quantify the robustness of causal estimates to violations of key assumptions and provide bounds on the magnitude of bias from specific sources. Since all causal inferences rest on untestable assumptions, such as no unmeasured confounding, correct model specification, or instrument validity, assessing how conclusions change under plausible deviations is essential for distinguishing robust causal findings from those highly sensitive to bias (VanderWeele and Ding, 2017). Sensitivity analyses directly address the classical epidemiological concern: how strong would an unmeasured confounder, selection mechanism, or measurement error process need to be to overturn our conclusions? This question is fundamental to honest causal inference, as it acknowledges the inherent uncertainty in observational studies while providing quantitative guardrails for interpretation. *E*-values, for instance, measure the minimum strength of association that an unmeasured confounder would need to have with both exposure and outcome to nullify the observed effect. In a study estimating the causal effect of fiber intake on gut microbial diversity, the *E*-value was 2.8, indicating that an unmeasured confounder would need to double the odds of both high fiber consumption and high diversity to explain away the result (Liu, 2025). Other sensitivity tools include placebo tests (e.g., testing for effects on unrelated outcomes), falsification endpoints, and bounds analysis under partial identification. Together, these techniques enhance the credibility of causal claims by making assumptions explicit and testing their resilience.

Moreover, software and workflow standardization play a critical role in reproducibility. The use of containerized environments (e.g., Docker, Singularity), version-controlled code repositories (e.g., GitHub), and workflow managers (e.g., Nextflow, Snakemake) ensures that analyses can be rerun exactly as performed. Initiatives like the NIH Cloud Platform Interoperability Effort are further enabling seamless deployment of causal ML pipelines across cloud infrastructures, lowering barriers to replication.

**Figure 4:**
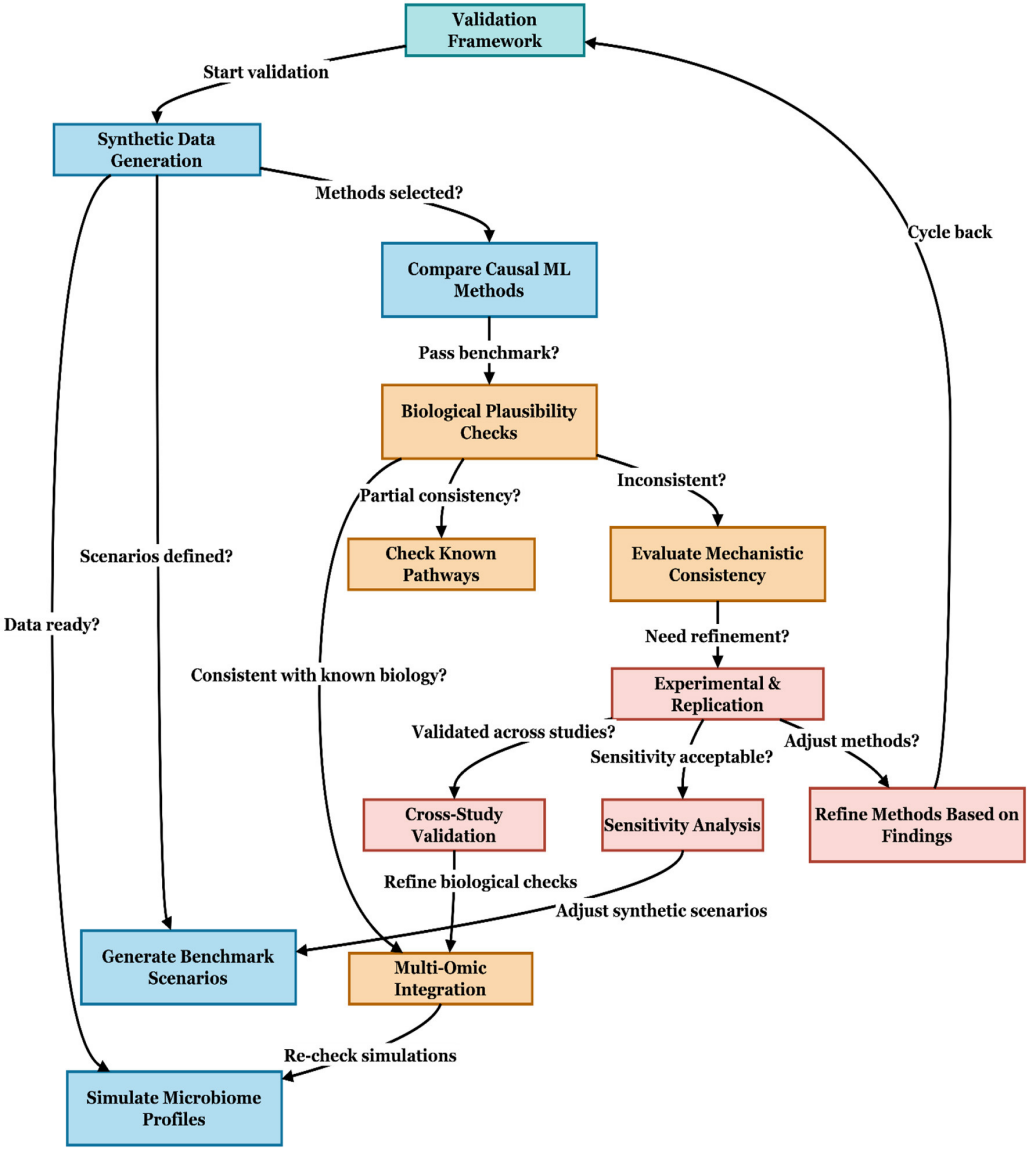
Validation framework for assessing causal inference methods in microbiome research.

The proposed validation framework (Figure 4) outlines a structured, iterative workflow for ensuring the robustness of causal machine learning applications in microbiome research, and summarizes the founding of Table 4. The process begins with synthetic data generation, enabling controlled scenarios for benchmarking. Candidate causal ML methods are then compared against established benchmarks. If performance thresholds are not met, the process cycles back for refinement. Next, biological plausibility checks assess whether the inferred relationships align with known pathways and mechanistic consistency. Where partial or inconsistent results arise, methods are directed to pathway validation or mechanistic evaluation, respectively. Validated models progress to experimental replication and cross-study validation, ensuring reproducibility across independent datasets. At this stage, sensitivity analyses further test the stability of results under varying assumptions. If methods require refinement, findings feed back into synthetic scenario adjustment or biological check refinement. The framework also incorporates multi-omic integration and microbiome profile simulation to strengthen biological credibility and facilitate re-checking of simulations. Finally, methods are iteratively refined based on findings, ensuring a continuous improvement loop. This workflow emphasizes not only benchmarking and reproducibility but also biological grounding and iterative refinement, making it well-suited for translating microbiome causal inference into policy-relevant insights. Having established the mechanistic plausibility and statistical robustness of causal ML methods, we now turn to the imperative of transparent reporting.

**Table 4:** Comparative overview of validation strategies in causal microbiome research, highlighting the purpose, tools, strengths, limitations, and examples of each strategy.

**Validation strategy**	**Purpose**	**Key tools/methods**	**Strengths**	**Limitations**	**Example in microbiome research**	**Reference(s)**
Synthetic data generation	Evaluate method performance under known causal structures	Microbiome Benchmark, GLASSO-sim, ARTIMIS, log-contrast models	Enables ground-truth evaluation; customizable; detects overfitting and spurious associations	Assumes simplified biology; results depend on simulation design	Testing Double ML vs. TMLE under high-dimensional microbiome networks	Clark et al., 2021; Krajewski and Hudgens, 2024
Multi-omic integration	Assess biological plausibility of inferred causal links	Metagenomics + metabolomics, structural equation modeling, mediation analysis	Links microbial taxa to functional outputs (e.g., metabolites)	Requires matched multi-omic datasets; expensive and complex	*Clostridium scindens* linked to reduced atherosclerosis via bile acids	Bing et al., 2022; Chetty and Blekhman, 2024; Mohammadi-Shemirani et al., 2023
Experimental validation	Confirm causality in controlled biological systems	Gnotobiotic animal models, *in vitro* co-cultures, CRISPR knockouts	Gold standard for mechanistic validation; closest to proof of causality	Resource-intensive; limited scalability and human generalizability	*Lactobacillus reuteri* predicted to suppress inflammation validated in gnotobiotic mice	Koch and Röhmel, 2004; Thomas et al., 2012; Walter et al., 2020
Cross-study validation	Test generalizability and robustness across independent datasets	Harmonized taxonomic profiling, meta-analysis, replication studies	High external validity; detects population- or cohort-specific effects	Limited by data availability, heterogeneity in measurement and populations	Causal effect of *Prevotella copri* on rheumatoid arthritis replicated across cohorts	Duvallet et al., 2017; Sinha et al., 2017; Smith et al., 2025
Sensitivity analysis	Quantify robustness to violations of untestable assumptions	E-values, placebo tests, falsification endpoints	Makes assumptions explicit; easy to implement; interpretable	Does not prove causality; provides bounds, not definitive answers	*E*-value of 2.8 for the causal effect of fiber intake on microbiome diversity	Chung and Chung, 2023; VanderWeele and Ding, 2017

## 5 STROBE-CML reporting guidelines

**Figure 5:**
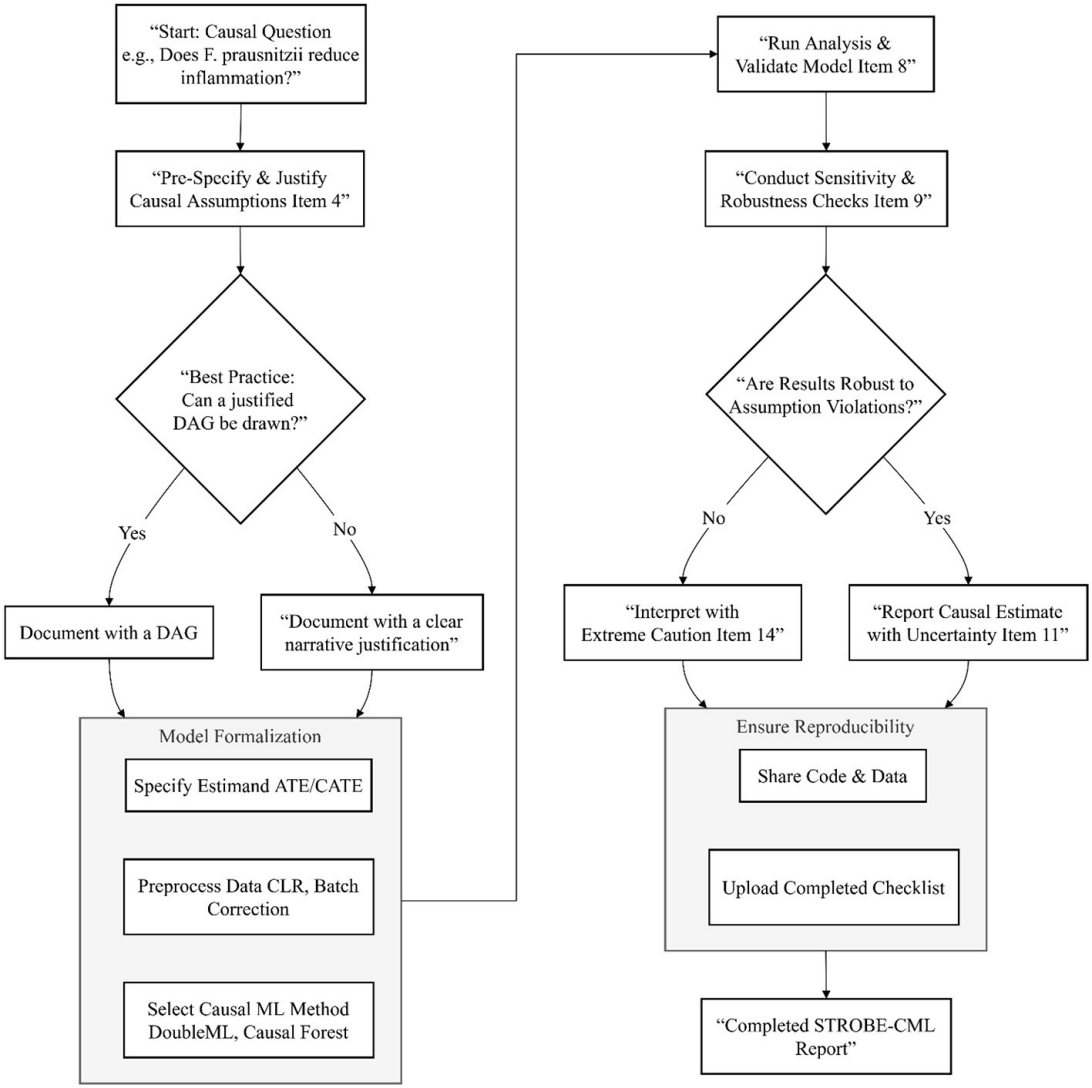
STROBE-CML reporting workflow. A practical workflow for implementing the STROBE-CML reporting guideline. The process begins with formalizing the causal question and, crucially, pre-specifying and documenting the underlying causal assumptions, ideally with a Directed Acyclic Graph (DAG). The analysis proceeds through model formalization, validation, and mandatory sensitivity checks to assess robustness to violations of those assumptions. The final, essential step is ensuring full transparency by sharing all materials, including a completed checklist.

**Figure 6:**
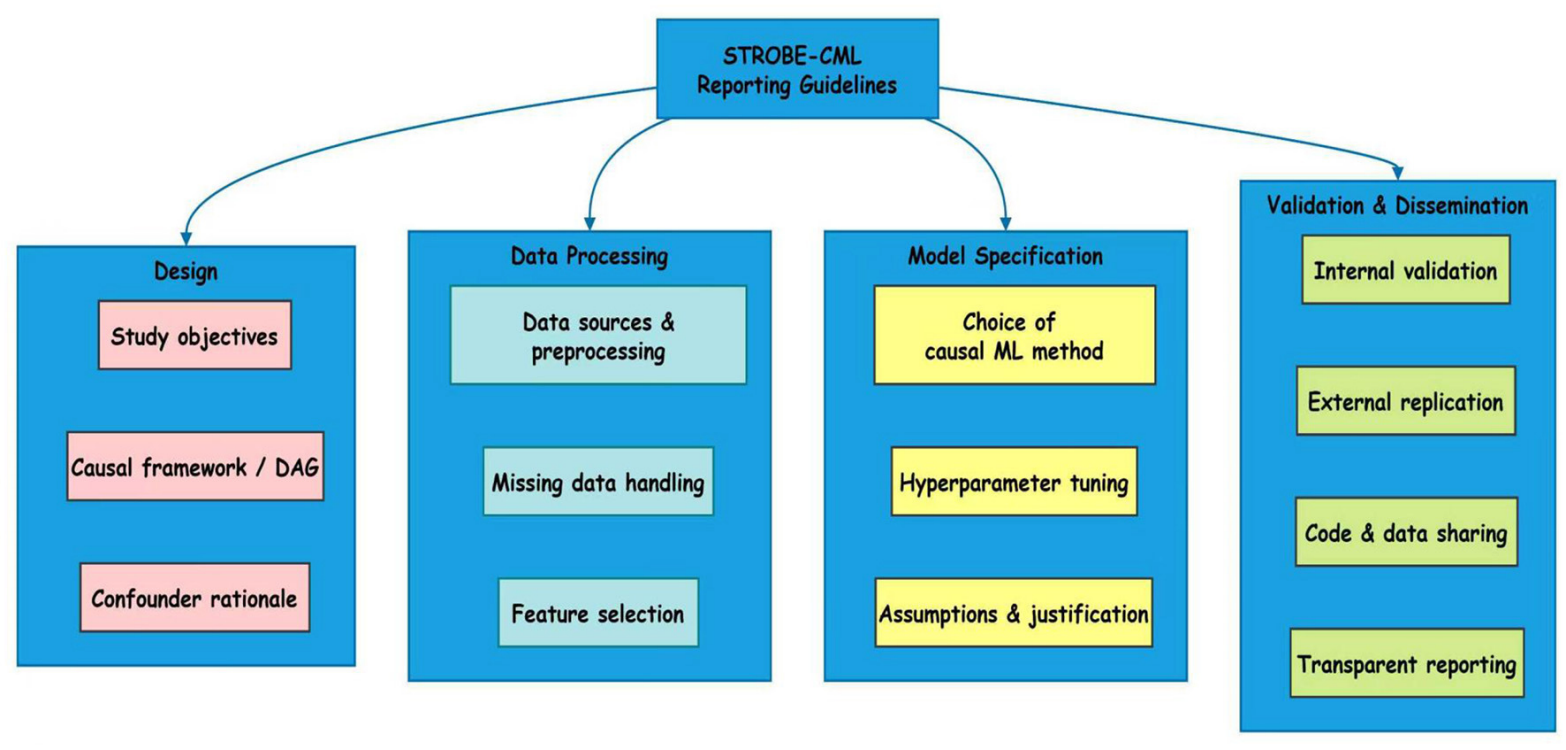
STROBE-CML Reporting guidelines for causal microbiome research.

To address the lack of standardization in causal machine learning applications, we propose the STROBE-CML (Strengthening the Reporting of Observational Studies in Epidemiology—Causal Machine Learning) guidelines (as illustrated in Figures 5, 6). This extension of the original STROBE statement incorporates elements specific to causal inference and ML methodology, ensuring that studies are reported with sufficient detail to allow critical appraisal, replication, and synthesis. The framework consists of 18 items organized across four domains: study design, data processing, model specification, and validation and dissemination.

Under data processing transparency, researchers must report all preprocessing steps in detail, including normalization methods (e.g., centered log-ratio transformation for compositional data), batch correction techniques (e.g., ComBat), and strategies for handling zero-inflation and rare taxa (Marcos-Zambrano et al., 2021). Given that these choices can profoundly influence downstream causal estimates, their documentation is non-negotiable. For example, improper handling of compositionality can induce spurious correlations, leading to biased causal inferences.

In model specification, full disclosure of algorithms, hyperparameters, and training procedures is required. This includes specifying whether cross-validation was used, how hyperparameters were tuned, and whether data splitting respected temporal or hierarchical structure (e.g., not splitting within subjects in longitudinal studies). The rationale for choosing a particular causal ML method, such as Double ML over propensity score matching, should be justified based on data characteristics and causal assumptions. Additionally, any software packages, versions, and random seeds must be reported to ensure reproducibility (Koh et al., 2025; Qiu et al., 2024).

For validation and dissemination, the guidelines mandate the reporting of both internal and external validation results. Internal validation should include sensitivity analyses (e.g., *E*-values, unmeasured confounding bounds), while external validation requires evidence of replication in independent datasets when available. Most critically, code and data availability must be ensured through public repositories such as Zenodo, Figshare, or GitHub, with processed data shared in standardized formats (e.g., BIOM, HDF5). Raw sequencing data should be deposited in INSDC-compliant databases (e.g., SRA, ENA). Exceptions for privacy-sensitive data should be accompanied by clear data access procedures.

The adoption of STROBE-CML (Table 5 and Figure 5) would significantly elevate the quality of causal microbiome research. Analogous extensions, such as STROBE-ME (for molecular epidemiology) and RECORD (for registry-based studies), have proven effective in improving reporting quality in their respective domains. We anticipate that STROBE-CML will serve a similar function, foster accountability and enabling meta-analytic synthesis of causal findings.

**Table 5:** STROBE-CML: proposed reporting guidelines for causal machine learning in microbiome research.

**Domain**	**Item**	**Checklist item**	**Description and rationale**	**Example for microbiome studies**
Title and Abstract	1	State the use of a causal machine learning method	Allows for immediate identification of the methodological framework	“Causal Forest model identifies *Akkermansia muciniphila* as a putative causal driver of improved insulin sensitivity”
	2	Specify the causal estimand of interest (e.g., ATE, CATE)	Clarifies the target of inference (e.g., population-average vs. heterogeneous effects)	“We estimated the Conditional Average Treatment Effect (CATE) of antibiotic exposure on *C. difficile* colonization risk”
Introduction	3	Formulate the research question using counterfactual or interventionist language	Ensures the objective is framed as a causal inquiry from the outset	“What is the expected change in inflammation if the abundance of *Faecalibacterium prausnitzii* were experimentally increased?”
	4	Justify and document the core causal assumptions. A Directed Acyclic Graph (DAG) is the recommended tool	Makes assumptions explicit. A DAG is best practice, but if the true structure is highly uncertain, a narrative justification and sensitivity analysis are required	“Primary assumption: the relationship between microbiome feature X and outcome Y is confounded by age, diet, and medication (visualized in DAG, Fig Sxx). Key uncertainty: potential for unmeasured confounding by physical activity was assessed via sensitivity analysis (Item 9)”
Methods	5	Report specific data preprocessing steps (normalization, batch correction, zero handling)	Preprocessing choices can dramatically alter compositional data and introduce severe bias	“We applied a centered log-ratio (CLR) transformation. We used the ConQuR tool for batch correction across sequencing runs”
	6	Justify the set of adjusted variables based on the stated causal assumptions (Item 4)	Prevents *ad-hoc* variable selection. Justification must be based on prior knowledge and the chosen identification strategy	“We adjusted for age, sex, and BMI, as our DAG (Figure Sxx) identifies this set as sufficient to block all backdoor paths between the microbiome feature and the outcome”
	7	Specify the Causal ML method, software, version, and all hyperparameters (with justification)	Ensures the analysis can be replicated	“We used the DoubleML package (v0.14.1) with a partially linear model. Nuisance functions were estimated with Random Forests (500 trees, max_depth = 5)”
	8	Detail the model validation protocol, including the cross-fitting strategy and performance of any nuisance models	Cross-fitting is essential for valid inference. Assessing nuisance model performance checks for practical violations of assumptions	“We used fivefold cross-fitting; the propensity score and outcome models achieved AUC > 0.7 and *R*^2^ > 0.3 on holdout folds, respectively”
	9	Conduct and report sensitivity analyses for the core causal assumptions, particularly for unmeasured confounding	This is non-negotiable. It quantifies the robustness of the finding and directly addresses the uncertainty documented in Item 4	“The *E*-value for our primary ATE was 2.8. A sensitivity analysis relaxing the exclusion restriction in our IV model showed results were robust (Supplementary Table xx)”
	10	For IV methods, report instrument strength (F-statistic) and tests for validity (e.g., pleiotropy)	Validates the core assumptions of the IV design	“Our genetic instrument had an F-statistic of 22.1. The MR-Egger intercept test (*p* = 0.35) did not suggest directional pleiotropy”
Results	11	Present causal estimates with appropriate uncertainty intervals (e.g., 95% CI)	Emphasizes that the output is a causal contrast with associated statistical uncertainty	“Increasing *Bifidobacterium* spp. abundance by 1 SD was associated with a 0.3 SD decrease in CRP (95% CI: −0.5, −0.1)”
	12	Visualize key results, including the distribution of treatment effects (if CATEs) and findings from sensitivity analyses	Makes complex results accessible. Showing the range of CATEs is a key advantage of Causal ML	Include in figures: a plot of the primary causal estimate (Figure xx), a histogram of CATEs from a Causal Forest (Figure xx), and a plot from the *E*-value sensitivity analysis (Figure xx)
Discussion	13	Interpret findings in light of biological plausibility and external evidence	Connects the statistical causal estimate to the underlying biology	“Our finding aligns with known butyrate production by *F. prausnitzii*, supporting biological plausibility”
	14	Acknowledge limitations, explicitly linking them to the causal assumptions in Item 4 and their sensitivity tests in Item 9	Demonstrates a coherent understanding of the study's validity, from assumptions to tests	“As noted in our DAG, we cannot rule out unmeasured confounding by detailed dietary components, though our *E*-value of 2.8 suggests robustness to a moderate confounder”
Transparency	15	Share analysis code in a public, version-controlled repository (e.g., GitHub)	Non-negotiable for reproducibility of complex Causal ML workflows	“Code available at: https://github.com/author/causal-microbiome-202YXXX”
	16	Share processed data in standardized formats (e.g., BIOM,.csv)	Allows for independent verification of the computational results	“Processed ASV table and metadata available in BIOM format via Figshare (doi: 10.xxx/yyyy)”
	17	For human data, describe access procedures if raw data cannot be shared	Ensures ethical compliance while maintaining transparency	“Raw sequencing data deposited in dbGaP (accession phs002345.vX.pX) under controlled access”
Overall	18	Include a completed STROBE-CML checklist as a supplementary file	Provides immediate verification of adherence and helps readers/reviewers quickly assess reporting completeness	“A completed STROBE-CML checklist detailing where each item is reported in the manuscript is provided as a supplementary file, Table xx”

This proposed extension of the STROBE statement, STROBE-CML (Strengthening the Reporting of Observational Studies in Epidemiology—Causal Machine Learning), provides 18 items to standardize reporting in causal microbiome research. It emphasizes transparency in assumptions, methods, validation, and data/code sharing. The guidelines support reproducibility and enable meta-synthesis of causal findings across studies.

## 6 Decision support tool for researchers

**Figure 7:**
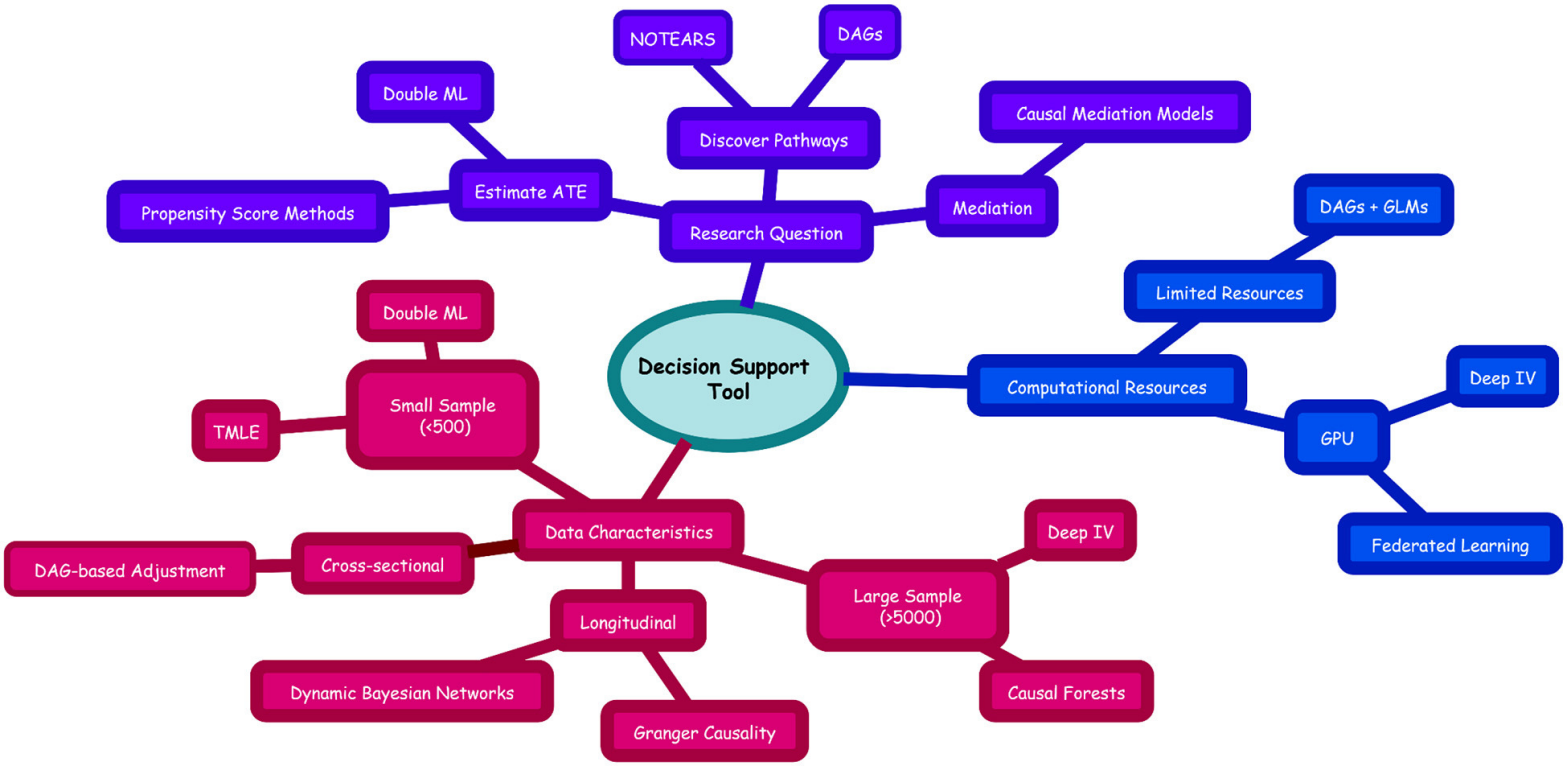
Decision support tool for causal inference in microbiome research. ATE, average treatment effect; Double ML, double machine learning; TMLE, targeted maximum likelihood estimation; DAG, directed acyclic graph; GLM, generalized linear model; Deep IV, deep instrumental variables; GPU, graphics processing unit.

Navigating the landscape of causal ML methods can be daunting, particularly for researchers without formal training in causal inference. To facilitate informed decision-making, we present a structured decision support tool that guides method selection based on three key criteria: data characteristics, research question, and computational resources (See Figure 7 for graphical illustration, and Table 1 in the Appendix S1 as a guide for researchers).

When considering data characteristics, sample size and dimensionality are paramount. For small to moderate sample sizes (< 500) with high-dimensional features (e.g., >1,000 ASVs), methods like Double ML or TMLE are preferred due to their double robustness and ability to handle confounder adjustment via machine learning. In contrast, for very large datasets (*n* > 5,000), deep learning–based approaches such as Deep IV, which uses neural networks to estimate non-parametric instrumental variable models, may offer superior flexibility in capturing complex, non-linear relationships (Koh et al., 2025). For longitudinal data, time-series methods like Granger causality or dynamic Bayesian networks are more appropriate than cross-sectional models.

The nature of the causal question further refines method choice. If the goal is to estimate the effect of a specific intervention (e.g., probiotic supplementation), methods that support average treatment effect (ATE) estimation, such as Double ML or propensity score weighting, are ideal. If the aim is exploratory, such as discovering causal pathways among microbial taxa and host metabolites, then structure learning methods like PC-algorithm or NOTEARS may be more suitable. For questions involving mediation (e.g., does microbial butyrate production mediate the effect of diet on inflammation?), causal mediation analysis with natural effect models is recommended.

Finally, computational resources and technical expertise must be considered. While Double ML is relatively accessible through packages like DoubleML in Python/R, methods like Deep IV require substantial GPU resources and deep learning expertise. Federated learning frameworks demand additional infrastructure for secure communication and model aggregation. For researchers with limited resources, simpler methods like DAG-based adjustment with generalized linear models may still yield valid inferences if assumptions are carefully evaluated.

This decision tool is implemented in the open-source platform Microbiome Causal Machine Learning (MiCML), which provides a user-friendly interface for selecting, applying, and validating causal ML methods (Koh et al., 2025). MiCML includes built-in benchmarking modules, sensitivity analysis tools, and automated reporting templates aligned with STROBE-CML, lowering the barrier to rigorous causal analysis.

## 7 Conclusion

Causal machine learning represents a paradigm shift in microbiome research, offering a principled pathway from observational associations to actionable causal insights. The methods reviewed here, ranging from Double ML and DAGs to federated learning and Mendelian Randomization, demonstrate the field's growing sophistication in addressing both classical epidemiological biases [confounding, selection, information bias, and collider bias] and unique computational challenges posed by high-dimensional, compositional microbiome data. However, methodological innovation alone is insufficient. Causal ML methods, regardless of sophistication, cannot overcome fundamental bias if core assumptions are violated or if study design is flawed (Hernán et al., 2004). Without standardized validation frameworks that explicitly evaluate bias mitigation, transparent reporting practices that make assumptions testable, and sensitivity analyses that quantify robustness to assumption violations, the risk of false discoveries and irreproducible findings remains high. The integration of causal inference principles with machine learning must be grounded in rigorous epidemiological thinking to ensure that computational power translates into valid causal knowledge rather than precisely estimated artifacts. This review has synthesized evidence from over 80 studies to advocate for a new standard of rigor in causal microbiome science. We emphasize the necessity of synthetic benchmarking to evaluate method performance, biological plausibility checks to ground findings in mechanism, and cross-study replication to ensure generalizability. Sensitivity analyses must become routine, making assumptions explicit and testing their impact on conclusions.

To institutionalize these practices, we propose the STROBE-CML reporting guidelines, which provide a comprehensive checklist for transparent, reproducible causal ML research. Coupled with the decision support tool and open-source implementation in MiCML, these recommendations offer a practical roadmap for researchers navigating the complexities of causal inference. As microbiome-based interventions advance toward clinical translation, the demand for credible causal evidence will only intensify. By adopting these frameworks, the scientific community can move beyond the reproducibility crisis and build a foundation of reliable, biologically meaningful knowledge that empowers both discovery and therapy.

## Data Availability

The original contributions presented in the study are included in the article/Supplementary material, further inquiries can be directed to the corresponding author.
