# Supplementary material for: STROBE-causal machine learning for the human microbiome: systematic review on methodological innovations and validation frameworks
Source: Front Microbiol. 2026 Mar 25;17:1705116. doi: 10.3389/fmicb.2026.1705116 (PMC13057537; doi:10.3389/fmicb.2026.1705116)
Supplement: Supplementary file 1 [file Table_1.docx]

APPENDIX

### **Comparative Table 1: Roles of Table 3, Table 4, and Table 5 in the Causal ML Workflow**

| Aspect | Table 3: Causal ML Methods | Table 4: Validation Strategies | Table 5: STROBE-CML Guidelines |
| --- | --- | --- | --- |
| Primary Purpose | Guide method selection based on research question and data type | Ensure credibility of causal claims through multi-level validation | Ensure transparency and reproducibility in reporting |
| When Used in Research Process | At the design and analysis stage | During validation and robustness testing | During write-up and submission |
| Key Question It Answers | Which causal ML method should I use? | How do I know my result is trustworthy? | What should I report so others can replicate and build on this? |
| Core Focus | Methodological diversity and assumptions | Robustness, biological plausibility, reproducibility | Completeness, openness, standardization |
| Audience Benefit | Helps researchers avoid methodological mismatch | Prevents overconfidence in unvalidated findings | Enables peer review, replication, meta-analysis |
| Link to Decision Tool (Section 6) | Direct input: method choice based on data characteristics | Direct input: validation plan (e.g., "Use synthetic data + E-values") | Output: ensures the final report meets best practices |
| Example Use Case | Choosing Double ML over TMLE for high-dimensional confounders | Validating a causal link with multi-omic data and cross-cohort replication | Reporting code, DAGs, and sensitivity analyses in the manuscript |
| Synergy with Other Tables | Feeds into validation (Table 4) and reporting (Table 5) | Tests the output of Table 3, informs Table 5 | Documents everything from Table 3 and Table 4 transparently |
| Analogy | A toolkit (which wrench to use) | A quality control checklist (did the repair hold?) | A service manual (how to document the repair for others) |

**Caption**:
This comparative table illustrates the complementary roles of the three core tables in this review. Together, they form an **end-to-end framework** for rigorous causal inference in microbiome research: **Table 3** enables appropriate method selection, **Table 4** ensures robust validation, and **Table 5** guarantees transparent, reproducible reporting. Their integration supports the STROBE-CML guidelines and the decision support tool in Section 6.
